# Genetic Diversity Analysis of Sugarcane Parents in Chinese Breeding Programmes Using gSSR Markers

**DOI:** 10.1155/2013/613062

**Published:** 2013-08-07

**Authors:** Qian You, Liping Xu, Yifeng Zheng, Youxiong Que

**Affiliations:** Key Laboratory of Sugarcane Biology and Genetic Breeding, Ministry of Agriculture, Fujian Agriculture and Forestry University, Fuzhou 350002, China

## Abstract

Sugarcane is the most important sugar and bioenergy crop in the world. The selection and combination of parents for crossing rely on an understanding of their genetic structures and molecular diversity. In the present study, 115 sugarcane genotypes used for parental crossing were genotyped based on five genomic simple sequence repeat marker (gSSR) loci and 88 polymorphic alleles of loci (100%) as detected by capillary electrophoresis. The values of genetic diversity parameters across the populations indicate that the genetic variation intrapopulation (90.5%) was much larger than that of interpopulation (9.5%). Cluster analysis revealed that there were three groups termed as groups I, II, and III within the 115 genotypes. The genotypes released by each breeding programme showed closer genetic relationships, except the YC series released by Hainan sugarcane breeding station. Using principle component analysis (PCA), the first and second principal components accounted for a cumulative 76% of the total variances, in which 43% were for common parents and 33% were for new parents, respectively. The knowledge obtained in this study should be useful to future breeding programs for increasing genetic diversity of sugarcane varieties and cultivars to meet the demand of sugarcane cultivation for sugar and bioenergy use.

## 1. Introduction

Sugarcane (*Saccharum *spp.) is the main sugar and bioenergy crop in the world. In comparison to other countries, Chinese sugar consumption is much lower and has only about 1/3 average of the world due to the different diet. However, the total sugar consumption, production, and import are in the second, third, and first positions in the world in recent years [1]. In addition, sugar from sugarcane occupies about 90%–92% of the total sugar output in China [2]. With an increasing demand for sugar, sugarcane shows more potential in China, leading to over one million sugarcane seedlings cultivated, which are produced from a total of 600–700 cross combinations every year in China [1]. The security of sugarcane cultivation is under threat from a number of diseases, especially smut disease caused by *Sporisorium scitamineum* and mosaic disease caused by *sugarcane mosaic virus* or *sorghum mosaic virus*. This leads to a demand for heterogeneity of cultivars. However, the heterogeneity of cultivars remains low, since the three “ROC” serial varieties account for about 85% of the total sugarcane cultivated area in China, with one (ROC22) responsible for about 50%–60% of the cultivated area in the last ten years [1]. Cross breeding is the most important way for breeding new sugarcane varieties and variety improvement, and it has played a significant role in the development of sugar industries in almost all the sugarcane-producing countries [3]. In addition, parental crosses of sugarcane always improve significantly the cane stalk yield and sugar content; thus, it is important to get the understanding of the genetic diversity of parents for crosses in breeding programs in China.

Traditional ways for sugarcane breeders to identify the relationships among varieties rely on anatomical and morphological characters [4]. In recent years, genetic diversity has been investigated for sugarcane cultivars or ancestral species by using several molecular methods, such as restriction fragment length polymorphism (RFLP) [5, 6], random amplified polymorphic DNA (RAPD) [7, 8], amplified fragment length polymorphism (AFLP) [9], intersimple sequence repeats (ISSR) [10, 11], sequence-related amplified polymorphism (SRAP) [12, 13], target region amplification polymorphism (TRAP) [14, 15], genomic in situ hybridization (GISH) [16, 17], fluorescence in situ hybridization (FISH) [17–19], genomic simple sequence repeats (gSSR, hereinafter referred to as SSR) [9], and expressed sequence tag-SSR (EST-SSR) markers [20]. Among all the above molecular techniques, SSR markers are widely used in the genetic diversity analysis of sugarcane because they are codominantly inherited, abundant, and highly reproducible [20–22]. Cordeiro et al. (2003) used six gSSR markers to assess the genetic diversity level between the 66 accessions which included the genera Saccharum (*S. officinarum*, *S. spontaneum*, and *S. sinense*), Old World *Erianthus* Michx. sect. *Ripidium*, North American *E. giganteus* (*S. giganteum*), *Sorghum,* and *Miscanthus *[23]. Liu et al. (2011) and Pan (2010) used polymorphic SSR DNA markers to genotype sugarcane clones with a fluorescence electrophoresis (CE)-based genotyping system [24, 25]. A few studies have also been reported on the genetic diversity of sugarcane parental accessions by SSR markers [26, 27]. 

Some accessions have played a particular key role in the development of commercial sugarcane varieties and thus have been designed as common breeding parents [28, 29]. In addition, new parental materials are more important for broadening genetic basis in the development of modern varieties used for cultivation and breeding [30, 31]. Therefore, investigation of the genetic relationships among common and new parental accessions is necessary for future sugarcane improvement and breeding in China.

In sugarcane breeding programmes, the choice of parents for crossing largely depends on the aims and objectives of the breeder. In the past, this was generally based on phenotypic and genotypic expression of the characters they display and especially on the superior progeny, that is, the potential ability of cane sugar yield of varieties derived from the cross combinations, which is also influenced by the environment and a series of uncontrolled factors. The objective of the present study is to evaluate the genetic diversity of 115 sugarcane cross parents, termed as common or new parents, using SSR markers. For the molecular analysis, two levels of analysis were investigated. Firstly, the within and between population diversity was evaluated on 64 common parents and 51 new parents, each represented by different groups, and the genetic parameters between the two groups of accessions were analyzed, respectively. Secondly, cluster analysis by unweighted pair group method with arithmetic mean (UPGMA) and principle component analysis (PCA) of 115 parents was performed. The information obtained in this study will be valuable for choice of parents and cross prediction and especially for the development of cultivar improvement programs in modern sugarcane breeding.

## 2. Materials and Methods

### 2.1. Plant Materials

The background of the sugarcane parents used in this study was given in Table 1. Leaf samples of a total of 115 sugarcane accessions, including 64 common parents and 51 new parents, were collected. They were cultivated in Sugarcane Resources Nursery of FAFU (Fujian Agriculture and Forestry University, Fuzhou, China) and Ruili Breeding Station in Yunnan Academy of Agriculture Science (Ruili, Yunnan, China).

### 2.2. DNA Extraction

DNA extractions from the leaf tissues were conducted according to biospin plant genomic DNA extraction kit specification (Bioflux, Japan). Each leaf sample was collected from three independent sugarcane plants and only +1 leaf from each plant. After detection of the quality and concentration, this batch of genomic DNA was diluted to a suitable concentration and stored at −20°C.

### 2.3. SSR Analysis

A total of five highly polymorphic SSR DNA markers (SMC334BS, SMC336BS, SMC36BUQ, SMC286CS, and SMC569CS) were selected from 221 ICSB sugarcane SSR markers [24, 32]. Forward primers of all these SSR primers were labeled with FAM, the fluorescence dye. PCR amplification was performed in a 25 *μ*L reaction containing 50 ng of genomic DNA, 2.5 *μ*L 10 × PCR buffer, 0.2 *μ*M of each primer, 200 *μ*M dNTP mixtures, and 1.0 U of *rTaq* polymerase. PCR comprised the following steps: the first cycle was preceded by a 3 min denaturation at 94°C, then thirty-one PCR cycles were performed in a PCR amplifier (Eppendorf 5333), with each cycle consisting of denaturation at 94°C for 30 s, annealing at either 58°C, 60°C, 62°C, or 64°C for 30 s (SMC286CS, SMC334BS, SMC569CS, and SMC36BUQ) and 62°C for 35 s (SMC336BS), and extension at 72°C for 30 or 35 s, and the last cycle was followed by a 2 min final extension at 72°C. Fragment analyses of amplified PCR products were conducted by capillary electrophoresis (CE) on ABI PRISM 377-96 DNA sequencer (Applied Biosystems) according to the manufacturer's instructions. Each CE sample included 1.0 *μ*L post-PCR reaction mixture, 0.5 *μ*L of ROX-360 size standards, and 8.5 *μ*L loading buffer of which the major ingredient contained polyacrylamide and dextran-blue. Then, PCR-amplified SSR DNA fragments were separated, and both the size standard and PCR amplified fragments were recorded automatically into individual GeneScan files.

### 2.4. Data Analyses

The data obtained from GeneScan files were analyzed with GeneMapper software (Applied Biosystems) to produce capillary electropherograms of amplified DNA fragments. GeneMapper parameters were set as follows: plate check module: Plate Check A; prerun module: GS PR36A-2400; run module: GS run 36A-2400; collect time: 2.5 h; and lanes: 64. An SSR allele or peak was scored either as present (1) or absent (0), except for “stutters,” “pull-ups,” “dinosaur tails,” or “minus adenine” [24, 32]. The polymorphic information content (PIC) was calculated by the formula PIC = 1 − ∑*P*_*i*_^2^, where *P*_*i*_ is the frequency of the population carrying the *i*th allele, counted for each SSR locus [21]. Then, the binary data matrices were used for genetic diversity parameter analysis. POPGENE 1.31 [33] was used to determine number of polymorphic bands (NPB); percentage of polymorphic bands (PPB); observed number of alleles (Na); and effective number of alleles (Ne). Nei's genetic diversity (*h*), mean values of total gene diversity (Ht), and Shannon's information index (*I*) were computed for each population based on allele frequencies and calculated for haploid data. In addition, gene diversity within populations (Hs), gene diversity between populations (Dst) by the formula (Dst = Ht − Hs), gene differentiation coefficient (Gst) calculated as (Ht − Hs)/Ht, and estimates of gene flow (Nm) were obtained by (1 − Gst)/2Gst. Based on Nei's (1978) genetic distances, a dendrogram showing the genetic relationships between genotypes was constructed by the unweighted pair group method with arithmetic average (UPGMA) using the NTSYS-pc version 2.1 [34, 35]. To further assess the genetic relationships between all of the accessions (9 series), PCA was performed based on genetic similarity using NTSYS-pc version 2.1 [35].

## 3. Results and Analysis

### 3.1. SSR Markers

SSR markers were utilized to assess genetic diversity among all the 115 sugarcane parental accessions in this study, and the major values of genetic diversity parameters derived were showed in Table 2.

A total of five SSR loci were used to evaluate 115 sugarcane accessions. Distinct fragments in the size ranging from 101 bp to 238 bp were scored for analysis. The major allele of five SSR loci was observed at the sizes of 147 bp, 168 bp, 122 bp, 146 bp, and 220 bp, with the ratio of 66.1%, 59.1%, 39.1%, 46.1%, and 66.1% with the primers SMC334BS, SMC336BS, SMC36BUQ, SMC286CS, and SMC569CS, respectively. A total of 88 alleles within the data set were obtained, and alleles per locus ranged from 11 to 26, with an average of 17.6. The average number of rare alleles produced in a single individual was 9.2 (range 6–15). The highest number of alleles was scored at locus SMC336BS (26 alleles). The PIC values of five SSR loci ranged from 0.753 to 0.897 with a mean value of 0.837. The PIC value of the SMC336BS locus was the highest (0.897), while the lowest (0.753) was observed from SMC36BUQ locus.

### 3.2. Genetic Diversity among 64 Common Parents, 51 New Parents, and All 115 Parents

Significant genetic variation was found among all 115 parents with the genetic similarity (GS) value ranging from 0.725 to 1.000. The GS value ranged from 0.730 to 1.000 within the group of 64 common parents and from 0.722 to 0.943 within the group of 51 new parents. Of note, the GS value was 1.000 between MT90-55 and HoCP93-750, indicating that there was no genetic dissimilarity between the two parents based on the five SSR loci.

Genetic parameters for the five microsatellite loci in the two groups, common parents and new parents, were given in Table 3. A total of 88 polymorphic bands within the entire data set were scored, while taking the two groups considered separately, 82 of them were within the 64 common parents (93.18%), and 69 of them were within the 51 new parents (78.41%). Observed numbers of alleles (Na) were the same (2.000) in the two groups, and effective numbers of alleles (Ne) were higher in new parents group (1.359) than in common parents group (1.302). Nei's gene diversity (*h*) was 0.178, and Shannon's information index (*I*) was 0.288 in the overall sugarcane testing accessions. In contrast to the total diversity, both sugarcane parent groups of common parents and new parents had relatively high diversity, *h* = 0.190 and 0.223 and *I* = 0.308 and 0.356, respectively.

Table 4 summarized the genetic differentiation of sugarcane accessions from the two groups. The values of Ht and Dst were higher in new parents group (Ht = 0.214, Dst = 0.058) than those in common parents group (Ht = 0.190, Dst = 0.032), while the value of genetic diversity (Hs) within population was similar in two groups (0.158 for common parents group and 0.156 for new parents group), indicating that the genetic diversity of these two groups mainly existed within populations. The gene flow index (Nm) within groups showed that low gene flow (2.429 and 1.335, resp.) occurred in both groups, while the Gst was high in both groups—0.171 and 0.273, respectively. The gene flow between the two groups was much higher (Nm = 4.762) than those in both groups. This also indicated that the genetic variation mainly existed within populations. 

### 3.3. Genetic Relationships of 115 Sugarcane Parents

Nine series from 115 accessions sorted by institution-based breeding programme are shown in Table 5. According to the information indicated in Table 1, we assigned them as the following nine series: GT series (13) from Guangxi Sugarcane Institute; YT series (13) from Guangzhou Institute of Sugarcane and Sugar Industry; YC series (10) from Hainan Sugarcane Breeding Station; FN series (7) from Sugarcane Research Institute of FAFU; MT series (7) from Sugarcane Research Institute, Fujian Academy of Agricultural Sciences; HoCP series (4) from Sugarcane Research Unit, Houma, Louisiana, United States Department of Agriculture, USA; CP series (10) from Sugarcane Experiment Station, Canal Point, Florida, United States Department of Agriculture, USA; and “ROC” series (13) from Taiwan Sugar Corporation. The rest of the sugarcane parents included 37 accessions from several breeding institutions different from all the above eight and were termed as OTHER.

Genetic diversity parameters for the 5 microsatellite markers in the 9 sugarcane series were presented in Table 5, indicating that except the highest NPB value (62 stands for 70.45%) observed in OTHER series, the polymorphisms among eight determinate series were as follows: YT (50, 56.82%) > YC (49, 55.68%) > GT (47, 53.41%) > “ROC” (46, 52.27%) > MT (44, 50.00%) > FN (37, 42.05%) > CP (36, 40.91%) > HoCP (32, 36.36%). Observed numbers of alleles (Na) were higher in OTHER (Na = 1.705) and YT (Na = 1.568) series compared to those of the remaining seven series. Moreover, effective numbers of alleles (Ne) were also higher in OTHER (Ne = 1.290) and YC (Ne = 1.290) series compared to those of the remaining seven determinate series. Except OTHER series (*h* = 0.177, *I* = 0.278), both the gene diversity (*h*) and the Shannon information index (*I*) were higher in YC (*h* = 0.178, *I* = 0.275) series but lower in CP series (*h* = 0.136, *I* = 0.181).

**Figure 1:**
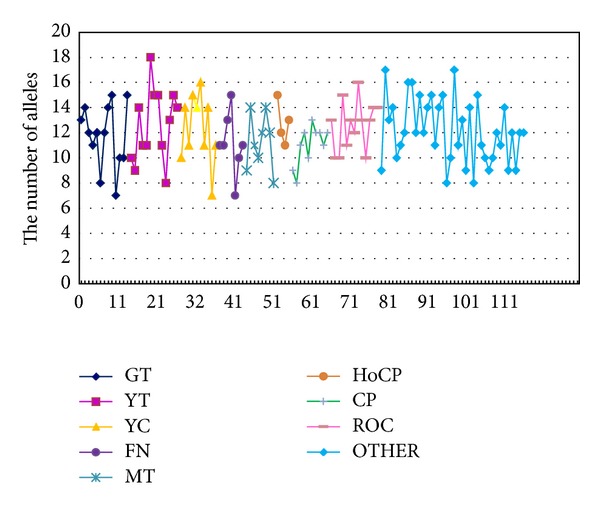
The number of alleles detected in 115 sugarcane accessions based on 5 SSR loci.

The number of alleles based on 5 SSR loci in different series of GT, YT, YC, FN, MT, HoCP, CP, “ROC,” and OTHER was illustrated in Figure 1. A total of 1,395 alleles were detected for all the 115 testing sugarcane accessions with an average of 12. The maximum number of alleles was 18 observed in YT93-159, while the minimum number was 7 in three accessions of GT90-55, YC96-48, and FN93-3608. Within the GT and FN series, the number of alleles both ranged from 7 to 15 with mean values of 11.8 and 11.1, respectively. In YT series, the number of alleles per locus ranged from 8 to 18 with an average of 12.6. Within MT series, the number of alleles ranged from 8 to 14, and the average number was 11.3. In HoCP series, the number of alleles was located between 11 and 15 with an average of 12.8. Within CP series, the number of alleles ranged from 8 to 13 with an average of 11.0. In “ROC” series, the number of alleles ranged from 10 to 16 with an average of 12.5. Within OTHER series, with an average of 12.0, the number of alleles was from 8 to 17.

### 3.4. Cluster Analysis

**Figure 2:**
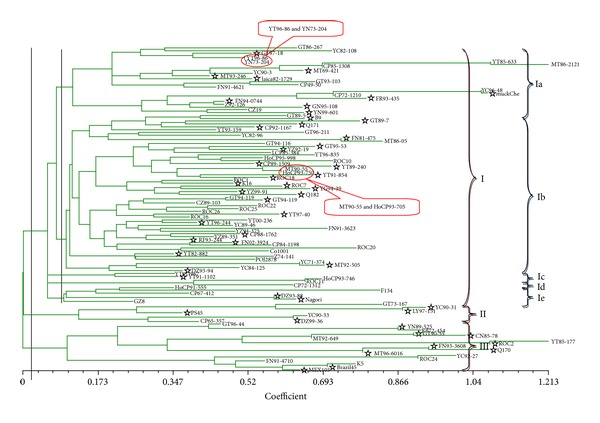
The UPGMA dendrogram of 115 Sugarcane parents based on 5 pairs of SSR primers.

The measure of genetic distance (GD) can be applied to any kind of organism without regard to ploidy or mating scheme [36], with genetic distance estimates hardly affected by the sample size [37]. Therefore, in this study, a UPGMA dendrogram was constructed based on Nei's genetic distance (Figure 2), showing the genetic relationships among the various series, including single series of GT, YT, YC, FN, MT, HoCP, CP, and “ROC” and complex series of OTHER and that between two groups of common parents (64) and new parents (51). The 115 sugarcane parents were classified into three groups (Group I, Group II, and Group III) at the level of GD = 0.03. Group I consisted of 53 common parents and 38 new parents, including 10 from GT, 12 from YT, 7 from YC, 5 from FN, 6 from MT, 4 from HoCP, 9 from CP, 11 from “ROC,” and 27 from OTHER. Group II contained 3 common parents and 4 new parents, including 1 from GT, 2 from YC, 1 from CP, and 3 from OTHER. Group III contained 8 common parents and 9 new parents, including 2 from GT, 1 from YT, 1 from YC, 2 from FN, 2 from MT, 2 from “ROC,” and 7 from OTHER. At the level of GD = 0.09, Group I could be further divided into five subgroups (Subgroup Ia, Ib, Ic, Id, and Ie). Ia contained 15 common parents and 8 new parents, including 3 from GT, 2 from YT, 3 from YC, 2 from FN, 3 from MT, 3 from CP, and 7 from OTHER. Ib consisted of 30 common parents and 27 new parents, including 5 from GT, 10 from YT, 4 from YC, 3 from FN, 3 from MT, 2 from HoCP, 4 from CP, 10 from “ROC,” and 16 from OTHER. Ic had only two parents from YT containing 1 common parent and 1 new parent. Id contained 3 parents from each of HoCP, CP, and “ROC” and belonged to common parents. Ie contained 4 common parents and 2 new parents, including 1 from HoCP, 1 from CP and 4 from OTHER series. 

It should be noted that Group I included most of the parents which came from different series. The above results demonstrate that the genotypes released by the same breeding institutions showed closer genetic relationships, except YC series released by Hainan sugarcane breeding station, which aimed at sugarcane germplasm innovation. It suggested that these parents should be useful in sugarcane cross breeding due to various genetic distances among them. Besides, a total of four testing accessions, including pairs of YT96-86 and YN73-204, plus MT90-55 and HoCP93-750, could not be distinguish based on the 5 microsatellite markers, and it may be due to their sharing of similar basis of genetic background.

### 3.5. Principal Component Analysis

**Figure 3:**
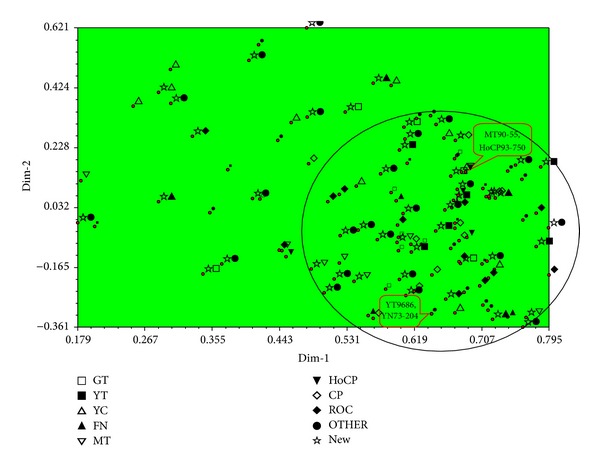
Principal coordinates analysis (PCA) of 115 sugarcane parents using 5 pairs of SSR markers based on genetic similarity.

PCA examined a dissimilarity matrix of pairwise differences between specimens and used eigenvalue analysis in order to take the variation between specimens and condense them into a limited number of dimensions. The maximum amount of variation was plotted as the first axis, with subsequent variation of lesser magnitude explained by each additional dimension [38]. The principal component analysis, which can be helpful for illustrating the genetic relationships of sugarcane parents as individual units, was calculated based on the SSR data matrix of the 5 loci for all 115 sugarcane accessions occupied in this study (Figure 3). The first and second principal components accounted for a cumulative 76% of the variance, including 43% for common parents and 33% for new parents, respectively. As shown in Figure 3, 115 sugarcane parents were scattered in a limited space, covering 90% of CP series, 85% of YT and “ROC” series, 77% of GT series, 75% of MT and HoCP series, 73% of OTHER series, 71% of FN series, and 50% of YC series, respectively. We found that the distribution of sugarcane accessions in CP, YT, and “ROC” series was relatively narrow, while it was wider in YC, FN, and OTHER series. This revealed that genetic basis of the latter group was more extensive than the former group. Furthermore, the plots of two pairs of sugarcane accessions (YT96-86/YN73-204 and MT90-55/HoCP93-750) overlapped strongly (Figure 3). This analysis could not differentiate YT96-86 from YN73-204 or MT90-55 from HoCP93-750 at least at a molecular level based on the 5 SSR markers used in this study.

## 4. Discussion and Conclusions

Improvement of sugarcane by genetic manipulation has been ongoing since 1888, following the observation in 1858 that sugarcane produced viable seed [1, 39]. According to the studies of Chen et al. (2011) and of Baver (1963), the contribution based on genetic improvement to increase the yield of cane sugar was estimated to be 75% of the yield increase attained by the Hawaiian sugar industry in the 1950s and more than 60% in the Chinese sugar industry in the last three decades [1, 40]. In Hawaii, the yield has improved every decade except in the 1970s, when disease problems plagued the sugar industry [40]. Although the degree to which varietal improvement has contributed to increase yield potential has varied widely from nation to nation, undoubtedly all nations have benefited to some degree by converting to newer, improved varieties from cross breeding. In addition, sugarcane is a potential bioenergy crop due to its high yield and high biomass. The world record and average in Hawaii (1978–1982) are 24.2 and 11.9 metric tons/ha/year, respectively. The 11.9 metric tons/ha/year represents a sugarcane dry matter yield of only 0.07 mt/ha/day, which is much lower than the theoretical maximum of 0.7 mt/ha/day estimated by Loomis and Williams [41]. 

In China, approximately 400 sugarcane varieties have been released in the last 50 years by cross breeding [42]. However, most of the sugarcane cultivars in the world can be dated back to only a few common ancestors [1, 19]. This may be due to the problem that the genetic basis of the sugarcane is limited; thus, new cultivars with interesting traits are difficult to be developed [43]. A similar situation has occurred in China, where the major cultivars in the 1980s, 1990s, and 2000s were ROC10, ROC16, and ROC22, respectively. Thus, till now, the heterogeneity of cultivars has been very low since the variety ROC22 takes about 50%–60% of the total sugarcane planting area. This limits any further increase of sugar yield per unit and has many potential risks of suffering from common diseases [1]. Sugarcane cross breeding largely depends on broadening the genetic basis and the selection of parents for crossing. The Hainan Sugarcane breeding station is responsible for sugarcane hybridization in China, innovation targets of parents, and introduction of new parents into sugarcane hybridization programs. An increase in the genetic diversity of parental accessions should be helpful to broaden the genetic basis of the sugarcane [26, 44].

In the present study, the genetic diversity of 115 sugarcane parents was evaluated based on 5 microsatellite loci. These SSR markers were highly robust and codominant as characterized by high PIC value (0.84 on average), but exhibited the lower level of polymorphism described by Liu (2011) who reported average PIC value = 0.70 [24]. However, the level of polymorphism obtained in our and Pan's studies was much higher than other SSR markers reported by Filho et al. (2010), who reported mean PIC value = 0.57 [45]. Genetic diversity of different series including eight determinate and one complex (OTHER) series showed that YC series had higher genetic diversity (*h* = 0.188 and *I* = 0.275) except OTHER (*h* = 0.177 and *I* = 0.278) and that CP and FN series had lower ones (*h* = 0.136 and 0.144, *I* = 0.181 and 0.219, resp.). This is consistent with the results reported by Li et al. (2005) and Lao et al. (2008) [46, 47].

In the present study, all 64 accessions in common parents group showed relatively lower diversity, compared with the higher diversity exhibited by 51 accessions of new parents group. The result was based on the value of Nei's genetic diversity (*h* = 0.190 < 0.223) and Shannon's information index (*I* = 0.308 < 0.356), indicating that the innovation of parents has showed a positive role in sugarcane breeding programs in China, since the group of new parents has higher genetic diversity, and thus, it will to some degree benefit the broadening of the genetic basis in sugarcane hybridization.

 The values of Nei's genetic diversity and Shannon's information index were much lower in other series than those in two groups. However, the level of diversity obtained in our research (two groups) was similar to previous research, which reported Nei's genetic diversity *h* = 0.222 and Shannon's information index *I* = 0.328 [13]. Since gene flow can resist the effect of genetic drift within populations and prevent the differentiation of populations with Nm > *l*, the genetic drift would lead to genetic differentiation among populations as the value of Nm < *l* [48]. The Nm value in this study was 4.762, indicating that there was no significant genetic differentiation between the two groups or nine series. The low genetic differentiation (Gst) among populations was primarily caused by the high level of gene flow. However, compared to wild sugarcane (Gst = 0.209) [13] and weedy rice (Gst = 0.387) [38], the Gst (0.095) of 115 sugarcane parents was still at a low level.

It is interesting that, in this study, both cluster and PCA analyses of individuals (including all the nine series) exhibited similar results: OTHER, YC, and GT series fell into three different groups and HoCP only belonged to Group I. Furthermore, a limited space covered 90% CP series, 85% YT and “ROC” series, and only 50% YC series, respectively. It was obvious that the distribution of accessions in CP, YT, and “ROC” series was relatively narrow while it was broader in YC, FN, and OTHER series. The results revealed that the genetic basis of YC, FN, and OTHER was more extensive than CP, YT, and “ROC” series, which also suggested that more attention should be made on the application of new parents in sugarcane hybrid breeding in the future. It was not difficult to find in the dendrogram (Figure 2) and PCA (Figure 3) that the clusters or components were closely related to their breeding institutions.

**Figure 4:**
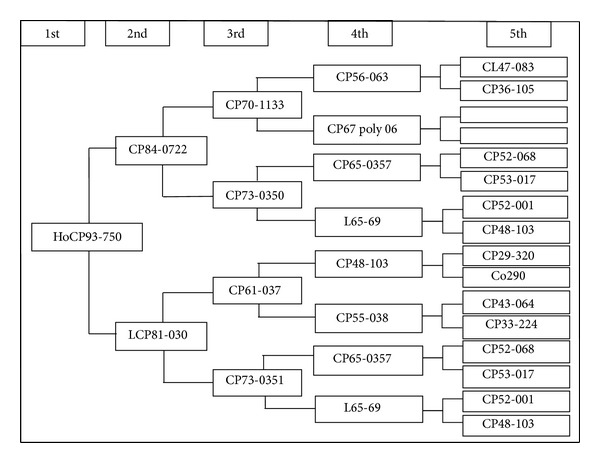
The pedigree of HoCP93-750.

**Figure 5:**
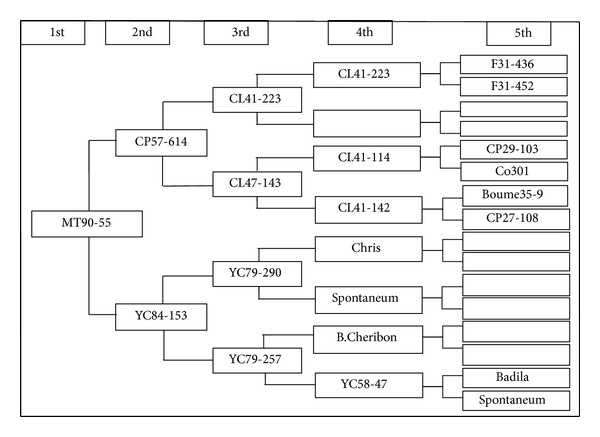
The pedigree of MT90-55.

It was also apparent that there were two pairs of four accessions (YT96-86 and YN73-204 at the level of GD = 0.50 and MT90-55 and HoCP93-750 at the level of 0.59) which the analysis failed to differentiate. Furthermore, the PCA analysis indicated that the plots of YT96-86 and YN73-204 or MT90-55 and HoCP93-750 overlapped entirely. This shows that the analysis could not differentiate between these accessions at the molecular level based on the five testing SSR loci and indicated that more SSR loci would be necessary for differentiation from MT90-55 to HoCP93-750 and from YT96-86 to YN73-204. For example, based on the pedigree, HoCP93-750 evolved from CP84-0722 and LCP81-030, while MT90-55 derived from CP57-614 and YC84-153 (Figures 4 and 5). From the pedigree of HoCP93-750 and MT90-55, it is obvious that we could not find the same parents between the two sugarcane clones within five generations. Therefore, it is inaccurate to analyze the genetic structures, genetic diversity, or genetic relationships only by pedigree records. If we want to further identify the four sugarcane clones, more SSR loci should be applied.

According to previous reports, gSSR markers produce polymorphisms based on the difference in the number of DNA repeat units in regions of the genome and derive from genomic DNA libraries at a high price, while EST-SSRs detect variations in the expressed portion of the genome and can be mined from the EST databases at low price [20, 49, 50]. EST-SSR technology has been widely used in many plants, such as rice [51], sorghum [52], wheat [53], and several other plant species. However, the usefulness of EST-SSRs varies in different varieties of sugarcane, as the level of polymorphism (PIC = 0.23) was lower than that of anonymous SSR markers (PIC = 0.72) in sugarcane cultivars. It was also reported that EST-SSRs had higher level of polymorphism across ancestral species (PIC = 0.66 > 0.62) [20]. In other research, the number of alleles of gSSRs loci (7–9) was more than EST-SSRs loci (4–6), and about 35% of the gSSRs had PIC values around 0.90 in contrast to 15% of the EST-SSRs (50). What should also be stressed is that the two types of SSR, gSSR and EST-SSR, made no significant difference at the average genetic similarity (GS) based on Dice coefficient and were in good agreement with pedigree information for genetic relationships analysis [50]. These results demonstrated that, in the future, EST-SSRs should be used together with gSSRs for genetic relationship analysis in sugarcane.

From the above discussion, identifying useful gSSRs is significant, but in sugarcane, this can be a lengthy and difficult process due to their complexity and their abundance within the sugarcane genome [20, 50, 54]. Therefore, there is further work required to promote this technique. This paper used only 5 pairs of gSSR primers in the genetic diversity analysis of 115 sugarcane parents in spite of the testing SSR loci being selected from a batch of gSSR loci (221 ICSB sugarcane SSR markers) and having shown to be robust and polymorphic. This suggests that more basic *Saccharum* species, more gSSR markers, and more molecular methods like EST-SSRs can be utilized in further study.

## Figures and Tables

**Table 1:** Description of the 115 sugarcane (*Saccharum *complex) accessions used in the SSR study.

Code	Name of accession	Collection place	Code	Name of accession	Collection place
1	GT86-267	FAFU	59	CP65-357	FAFU
2	GT89-5	FAFU	60	CP67-412	FAFU
3	GT93-103	FAFU	61	CP72-1210	FAFU
4	GT94-116	FAFU	62	CP72-1312	FAFU
5	GT94-119	FAFU	63	CP84-1198	FAFU
6	GT96-44	FAFU	64	CP85-1308	Ruili
7	GT96-211	FAFU	65	*CP88-1762	FAFU
8	GT73-167	Ruili	66	*CP89-1509	FAFU
9	*GT89-7	FAFU	67	*CP92-1167	FAFU
10	*GT90-55	FAFU	68	ROC1	FAFU
11	*GT94-119	FAFU	69	ROC10	Ruili
12	*GT95-53	FAFU	70	ROC11	Ruili
13	*GF97-18	FAFU	71	ROC16	FAFU
14	YT96-835	FAFU	72	ROC20	Ruili
15	YT96-86	FAFU	73	ROC22	Ruili
16	YT00-236	FAFU	74	ROC24	FAFU
17	YT85-633	Ruili	75	ROC25	Ruili
18	YT91-967	Ruili	76	ROC26	FAFU
19	YT93-159	Ruili	77	*ROC2	FAFU
20	YT85-177	Ruili	78	*ROC7	FAFU
21	*YT82-882	FAFU	79	*ROC18	FAFU
22	*YT89-240	Ruili	80	F134	Ruili
23	*YT91-854	FAFU	81	*DZ93-88	Ruili
24	*YT91-1102	FAFU	82	*DZ93-94	Ruili
25	*YT96-244	FAFU	83	*DZ99-36	Ruili
26	*YT97-40	FAFU	84	YZ89-351	FAFU
27	YC71-374	FAFU	85	YZ94-375	Ruili
28	YC82-96	FAFU	86	*YZ92-19	FAFU
29	YC82-108	FAFU	87	*YZ99-91	FAFU
30	YC84-125	FAFU	88	*Q170	FAFU
31	YC89-46	FAFU	89	*Q171	FAFU
32	YC90-3	FAFU	90	*Q182	FAFU
33	YC90-33	FAFU	91	*CZ89-103	Ruili
34	YC92-27	FAFU	92	CZ19	FAFU
35	YC96-48	FAFU	93	*CN85-78	FAFU
36	*YC90-31	FAFU	94	ZZ74-141	FAFU
37	FN91-3623	FAFU	95	ZZ92-126	FAFU
38	FN91-4621	FAFU	96	POJ2878	FAFU
39	FN91-4710	FAFU	97	Co1001	FAFU
40	*FN81-475	FAFU	98	RB72-454	Ruili
41	*FN93-3608	FAFU	99	K5	FAFU
42	*FN94-0744	FAFU	100	GZ8	FAFU
43	*FN02-3924	FAFU	101	LCP85-384	FAFU
44	MT86-05	FAFU	102	*Nagori	Ruili
45	MT86-2121	Ruili	103	*muck che	Ruili
46	MT90-55	FAFU	104	*laica82-1729	Ruili
47	MT92-649	FAFU	105	*YG94-39	FAFU
48	*MT69-421	Ruili	106	*RF93-244	FAFU
49	*MT92-505	FAFU	107	*Brazil45	FAFU
50	*MT93-246	FAFU	108	*FR93-435	FAFU
51	*MT96-6016	FAFU	109	*MEX105	FAFU
52	HoCP91-555	FAFU	110	*YZ99-601	FAFU
53	HoCP93-746	Ruili	111	*K16	FAFU
54	HoCP93-750	FAFU	112	*B9	FAFU
55	HoCP95-998	Ruili	113	*PS45	Ruili
56	YN73-204	FAFU	114	*LY97-151	Ruili
57	*YN89-525	FAFU	115	*GN95-108	FAFU
58	CP49-50	FAFU			

Sugarcane Resources Nursery of Fujian Agriculture and Forestry University (FAFU); Ruili Breeding Station in Yunnan Academy of Agriculture Science (Ruili); *represents new parents.

**Table 2:** The allele detection results of 5 SSR markers used for evaluation of 115 sugarcane accessions.

Primer name	Number of alleles	Number of rare alleles	Range of allele size (bp)	Major allele		PIC
				Size (bp)	Frequency (%)	
SMC334BS	19	6	136–169	147	66.10	0.889
SMC336BS	26	15	136–192	168	59.10	0.897
SMC36BUQ	11	8	101–147	122	39.10	0.753
SMC286CS	15	6	123–169	146	46.10	0.865
SMC569CS	17	11	159–238	220	66.10	0.779

Average	17.6	9.2				0.837

Rare allele means that the frequency of the allele is less than 5.0%; the major allele accounts for the highest proportion in all alleles.

**Table 3:** The values of genetic diversity parameters for sugarcane accessions of common and new parents in different groups, estimated based on polymorphisms of 5 SSR loci.

Group	Clones size	NPB	PPB (%)	Na	Ne	*h *	*I *
Common parents	64	82	93.18	2.000	1.302	0.190	0.308
New parents	51	69	78.41	2.000	1.359	0.223	0.356
Total	115	88	100.0	2.000	1.283	0.178	0.288

Number of polymorphic bands (NPB); percentage of polymorphic bands (PPB); observed number of alleles (Na); effective number of alleles (Ne); Nei's genetic diversity (*h*); Shannon's information index (*I*).

**Table 4:** Genetic diversity and differentiation of sugarcane accessions between common and new parents, estimated by POPGENE (version 1.31).

Group	Clones size	Ht	Hs	Dst	Gst	Nm
Common parents	64	0.190	0.158	0.032	0.171	2.429
New parents	51	0.214	0.156	0.058	0.273	1.335
Total	115	0.176	0.159	0.017	0.095	4.762

Mean values of total gene diversity (Ht), gene diversity within populations (Hs), gene diversity between populations (Dst), gene differentiation coefficient (Gst), and estimates of gene flow from Gst (Nm) were obtained by (1 − Gst)/2Gst.

**Table 5:** Genetic diversity of sugarcane parents in 9 series released by different breeding institutions, estimated based on polymorphisms of 5 SSR loci.

Series	Clones size	NPB	PPB (%)	Na	Ne	*h *	*I *
GT	13	47	53.41	1.534	1.262	0.162	0.250
YT	13	50	56.82	1.568	1.267	0.166	0.258
YC	10	49	55.68	1.557	1.283	0.178	0.275
FN	7	37	42.05	1.421	1.236	0.144	0.219
MT	8	44	50.00	1.500	1.259	0.161	0.247
HoCP	4	32	36.36	1.364	1.268	0.152	0.221
CP	10	36	40.91	1.409	1.222	0.136	0.181
ROC	13	46	52.27	1.523	1.259	0.160	0.247
OTHER	37	62	70.45	1.705	1.290	0.177	0.278
